# High expression of *THY1* is a prognostic marker for gastric Cancer: Deciphering its transcriptional regulation as a component of the Epithelial–mesenchymal transition

**DOI:** 10.1016/j.bbrep.2025.102050

**Published:** 2025-05-11

**Authors:** Paulo Rohan, Everton Cruz dos Santos, Pedro Leite Azevedo, Jessica Oliveira da Conceição, Eliana Abdelhay, Renata Binato

**Affiliations:** Stem Cell Laboratory, Division of Specialized Laboratories, Instituto Nacional de Câncer (INCA), Rio de Janeiro, 20230-130, RJ, Brazil

**Keywords:** Gastric cancer, THY1, Regulatory networks, Epithelial–mesenchymal transition, Bioinformatics

## Abstract

Gastric cancer (GC) remains one of the leading causes of cancer-related mortality worldwide, with high molecular heterogeneity contributing to its poor prognosis. Among potential biomarkers, *THY1* is associated with aggressive tumor behavior and poor patient outcomes. However, the transcriptional mechanisms governing *THY1* expression in GC remain largely unexplored. This study aimed to systematically investigate the upstream regulatory landscape of *THY1* and its role in tumor progression. By integrating multicohort transcriptomic data (n = 945), we inferred consensus transcriptional regulatory networks (TRNs) and identified six putative transcription factors (PRRX1, TWIST1, SNAI2, MEIS3, VENTX, and EGR2) as robust regulators of *THY1*. The functional enrichment analysis revealed that these regulators are involved in the epithelial–mesenchymal transition (EMT) and extracellular matrix remodeling, key processes associated with tumor invasion and metastasis. Experimental validation using chromatin immunoprecipitation (ChIP) assays indicated the direct and differential binding of TWIST1 and SNAI2 to the *THY1* promoter, supporting their roles as key regulators of *THY1* expression in GC. Our findings provide a mechanistic link between *THY1* expression and EMT transcriptional programs, offering insights into its association with a poor prognosis. By integrating bioinformatic predictions with experimental demonstration, this study not only improves our understanding of *THY1* regulation but also provides a framework for dissecting the transcriptional networks governing aggressive tumor phenotypes. These results contribute to a broader understanding of GC progression and may inform future therapeutic strategies targeting EMT-related pathways in *THY1*^high^ GC.

## Highlights

• TWIST1 and SNAI2 were validated as key regulators of *THY1* in gastric cancer.
• *THY1* is linked to the epithelial–mesenchymal transition in gastric cancer.
• Multicohort bioinformatics paired with experiments ensure robust findings.
• A framework for studying the transcriptional regulation of cancer biomarkers was revealed.

## 1 Introduction

Gastric cancer (GC) is a highly heterogeneous and aggressive malignancy and is the fifth leading cause of cancer-related death worldwide [1]. Despite advances in diagnostic techniques and therapeutic strategies, the prognosis of GC remains poor, with a five-year survival rate of less than 10 % in patients with advanced disease, which represents the majority of diagnosed cases [2,3]. One of the major challenges in improving patient outcomes is the molecular heterogeneity of GC, which influences tumor behavior, therapy resistance, and disease progression [4]. At the histological level, adenocarcinoma is the predominant type of GC, accounting for 95 % of all cases, and is divided into two main subtypes according to Lauren's classification—intestinal (IGC) and diffuse GC (DGC)—each exhibiting distinct etiological, epidemiological, and genetic characteristics [5,6]. To address this complexity, molecular subtyping and biomarker discovery have become key strategies for refining patient classification and identifying subgroups with distinct prognostic and therapeutic profiles [7]. Among potential biomarkers, *THY1* has emerged as a strong candidate because of its association with tumor aggressiveness and poor prognosis of GC [[8], [9], [10], [11], [12]]. However, the regulatory mechanisms governing *THY1* expression remain poorly understood, limiting our ability to determine its precise role in GC progression.

*THY1,* also known as CD90, is a glycosylphosphatidylinositol (GPI)-anchored cell surface glycoprotein that plays critical roles in cell adhesion, migration, and differentiation [[13], [14], [15]]. It is broadly expressed in neurons, thymocytes, fibroblasts, mesenchymal stem cells, and endothelial cells, where it modulates cell–cell and cell–matrix interactions through integrin-mediated signaling [[15], [16], [17]]. In cancer, particularly in carcinomas, *THY1* has a context-dependent role, acting as either a tumor suppressor or a promoter of tumor progression, depending on cellular and microenvironmental factors [[18], [19], [20], [21]]. In hepatocellular and renal carcinomas, *THY1* expression has been linked to cancer stem-like properties, enhanced invasiveness, and poor prognosis [18,19]. Similarly, in GC, high *THY1* expression correlates with aggressive tumor behavior, increased metastatic potential, and worse patient outcomes [[8], [9], [10], [11], [12]]. Additionally, *THY1*-expressing gastric cancer cells exhibit mesenchymal traits, increased adhesion to the extracellular matrix (ECM), and increased proliferative and migratory potential, facilitating tumor invasion and dissemination [[22], [23], [24]]. Moreover, *THY1*-positive GC tumor cells have been shown to resist conventional chemotherapy, reinforcing their roles in tumor progression and therapy resistance [25].

Despite its emerging role as a prognostic biomarker, the upstream regulatory mechanisms controlling *THY1* expression in GC remain largely unexplored. Previous studies have reported evolutionary divergence in the regulatory elements of *THY1* between humans and model organisms, complicating the development of models that accurately reflect human *THY1* regulation [26]. Furthermore, *THY1* expression is governed by distinct regulatory regions that vary across tissues, with deletions of these regions selectively abrogating expression in some tissues while sparing others [[27], [28], [29]]. Given this context-dependent regulatory complexity and its strong association with tumor aggressiveness, understanding the transcriptional regulation of *THY1* is essential for elucidating the molecular programs that drive invasion, metastasis, and therapy resistance. However, the precise mechanisms that control *THY1* expression in gastric cancer remain unclear. What triggers its upregulation in aggressive tumors, and what transcription factors dictate its expression? Addressing these questions is crucial for elucidating the role of *THY1* in tumor progression.

We systematically investigated the transcriptional regulation of *THY1* in GC using a multicohort integrative approach that combines computational modeling with experimental validation to address this challenge. Given the context-dependent nature of *THY1* expression and its established association with the EMT and tumor progression [12], we hypothesized that *THY1* is embedded within a transcriptional program that governs invasion and metastasis in GC. We inferred transcriptional regulatory networks (TRNs) across independent cohorts and identified candidate regulators that are consistently associated with *THY1* expression to test this hypothesis. We refined these findings by performing a correlation meta-analysis and predicting transcription factor-binding sites, allowing us to prioritize six putative transcriptional regulators of *THY1*. Finally, we validated these regulatory interactions by combining conservation analyses with chromatin immunoprecipitation (ChIP) assays, providing experimental evidence supporting the role of TWIST1 and SNAI2 as potential key regulators of *THY1* expression in gastric cancer cells. Together, our findings reveal the transcriptional network that could govern *THY1* expression, support a mechanistic link between *THY1* and the EMT, and provide a biological rationale for its association with a poor prognosis. By integrating *in silico* predictions with experimental validation, this study provides a framework for dissecting the regulatory landscapes of key prognostic markers in cancer.

## 2 Materials and methods

### 2.1 Data acquisition

Datasets comprising a total of 945 GC samples, including both RNA-seq and microarray data, along with their corresponding clinical information, were obtained from the Genomic Data Commons (GDC) portal and the Gene Expression Omnibus (GEO) database [[30], [31], [32]]. Data retrieval was performed using R version 4.4.2 (https://www.r-project.org/, accessed on January 10, 2025); R software, RRID:SCR_001905) along with Bioconductor (version 3.20 (http://www.bioconductor.org/, accessed on January 14, 2025); Bioconductor, RRID:SCR_006442), and the following packages were used: TCGAbiolinks (version 2.34.0; TCGAbiolinks, RRID:SCR_017683) for TCGA data and GEOquery (version 2.74.0; GEOquery, RRID:SCR_000146) for the GEO data [[33], [34], [35], [36]].

**Fig. 1:**
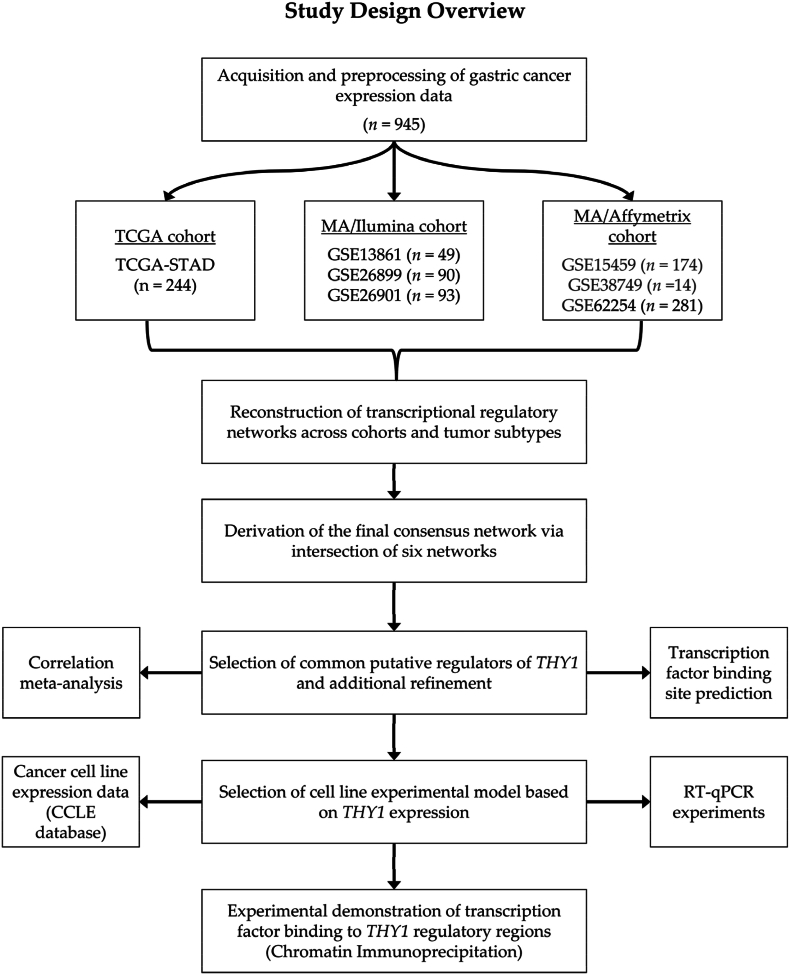
Study design**.** A total of 945 gastric cancer (GC) samples, including both RNA-seq and microarray data, were retrieved along with their corresponding clinical information. These datasets were grouped into three main cohorts: TCGA (n = 244), MA/Illumina (n = 232, combining GSE13861, GSE26899, and GSE26901), and MA/Affymetrix (n = 469, combining GSE15459, GSE38749, and GSE62254). Transcriptional regulatory networks (TRNs) were reconstructed for each tumor subtype (intestinal and diffuse) within each cohort. A final consensus network was derived by intersecting six subtype-specific TRNs across the cohorts. From this consensus network, common putative regulators of *THY1* were identified and refined using a correlation meta-analysis and transcription factor binding site predictions. The gastric cancer cell line expression data from the Cancer Cell Line Encyclopedia (CCLE) database were subsequently analyzed to select representative cell lines with high (*THY1*^high^) and low (*THY1*^low^) *THY1* expression. The selected cell lines were validated using RT‒qPCR, and a robust experimental model was established. Chromatin immunoprecipitation (ChIP) assays coupled with quantitative PCR (ChIP‒qPCR) were performed to assess the binding of transcription factors to *THY1* regulatory regions.

Among the 945 samples, 244 were from TCGA-STAD study, while the remaining 701 were derived from the following GEO studies: GSE13861 (n = 49), GSE15459 (n = 174), GSE26899 (n = 90), GSE26901 (n = 93), GSE38749 (n = 14), and GSE62254 (n = 281). Among these studies, GSE13861 used the Illumina HumanWG-6 v3.0 Expression BeadChip, and GSE26899 and GSE26901 used the Illumina HumanHT-12 V3.0 Expression BeadChip. GSE15459, GSE38749, and GSE62254 employed the Affymetrix Human Genome U133 Plus 2.0 Array. The data were then grouped into three main cohorts: TCGA (n = 244), MA/Illumina (GSE13861, GSE26899, and GSE26901; n = 232), and MA/Affymetrix (GSE15459, GSE38749, and GSE62254; n = 469), where MA stands for microarray. The design and workflow of this study are shown in Fig. 1.

### 2.2 Preprocessing and normalization

For TCGA cohort, we used the median-of-ratios (MRM) method and variance stabilizing transformation (VST) from the DESeq2 package (version 1.46.0; DESeq2, RRID:SCR_015687) to normalize the data [37]. For the microarray data, quantile normalization was applied to the MA/Illumina datasets, whereas the robust multichip average (RMA) method was employed for the MA/Affymetrix cohort using the limma package (version 3.62.2; limma, RRID:SCR_010943) [38]. Batch effect correction for the MA/Affymetrix and MA/Illumina datasets was performed using the sva package (version 3.20.0; sva, RRID:SCR_012836) [39].

### 2.3 Reconstruction of transcriptional regulatory networks

After normalization, we first identified the set of genes common to all three cohorts by constructing a Venn diagram. Next, we stratified each cohort into intestinal and diffuse subtypes to account for potential biases arising from different tumor subtypes. TRNs were then reconstructed for each subtype in each cohort using the RTN package (version 2.30.0) and the Algorithm for the Reconstruction of Accurate Cellular Networks (ARACne) [40,41].

We relied on the annotations available in the Animal Transcription Factor Database (AnimalTFDB) to identify transcription factors (TFs) [42,43]. We performed 1000 permutations to evaluate the statistical significance of the inferred regulatory interactions, applying a cutoff adjusted p value < 3.28 × 10^−7^. A bootstrap analysis with 1000 iterations was subsequently performed to assess the robustness of these interactions. A data processing inequality (DPI) algorithm was finally applied to remove indirect interactions.

Networks were constructed separately for each subtype in each of the three cohorts, and only the regulatory interactions common to all subtypes and cohorts were retained for downstream analyses. This approach minimized the influence of tumor subtype heterogeneity on the final set of regulatory relationships. Additionally, the generated networks were visualized using Cytoscape software (version 3.10.2; Cytoscape, RRID:SCR_003032) [44].

### 2.4 Correlation meta-analysis

Using the reconstructed TRNs, we first selected regulatory relationships involving the *THY1* gene. The Pearson correlation test was then performed between *THY1* and each of its predicted regulators in every cohort and subtype. The metafor package (version 4.6–0) was used to combine these correlations into a single estimate [45]. Specifically, each Pearson correlation coefficient was converted into an effect size via Fisher's z-transformation, and a random-effects model was subsequently fitted to obtain the pooled correlation coefficient and its confidence interval.

### 2.5 Transcription factor binding site prediction

We first retrieved the putative promoter region of the *THY1* gene from the Ensembl canonical transcript ENST00000284240.10 to identify potential binding sites for *THY1* regulators [46]. A 3-kb window upstream of the transcription start site was designated the putative promoter. Additionally, we obtained the first intron of *THY1* (2186 bp), which is known in the literature to be a regulatory region [27]. Next, we obtained the position weight matrices (PWMs) for the predicted *THY1* regulators from the JASPAR2024 package (version 0.99.6) [47]. Using these PWMs, we employed the Biostrings package (version 2.74.1; Biostrings, RRID:SCR_016949) to scan for corresponding binding sites within the 3-kb promoter region or the intron, applying a minimum score threshold of 85 % [48]. Only canonical binding sites meeting this threshold were extracted and considered for further analyses. The following consensus binding sites were used: MSC (MA0665.1; forward: AACAGCTGTT, reverse: AACAGCTGTT), SNAI2 (MA0745.3; forward: CACCTG, reverse: CAGGTG), TWIST1 (MA1123.3; forward: CATCTG, reverse: CAGATG), PRRX1 (MA0716.2; forward: TAATT, reverse: AATTA), EGR2 (MA0472.2; forward: CGCCCACGC, reverse: GCGTGGGCG), HEY1 (MA0823.1; forward: CACGTG, reverse: CACGTG), BCL6B (MA0731.1; forward: AATTCCTAGAAAGCA, reverse: TGCTTTCTAGGAATT), and MEIS3 (MA0775.2; forward: TGTCA, reverse: TGACA). For VENTX (MA0724.1), where a well-established consensus sequence was not observed, we used only the minimum score threshold of 85 %.

### 2.6 Transcription factor binding site conservation

We retrieved the 3-kb region upstream of the *THY1* transcription start site and the first intron for the following species evaluate the conservation of the canonical binding sites for the selected transcription factors: *Homo sapiens* (GCA_000001405.29), *Pan troglodytes* (GCA_000001515.5), *Pan paniscus* (GCA_000258655.2), *Gorilla gorilla* (GCA_000151905.3), *Cercocebus atys* (GCA_000955945.1), *Nomascus leucogenys* (GCA_000146795.3), *Papio anubis* (GCA_008728515.1), *Macaca fascicularis* (GCA_011100615.1), *Macaca nemestrina* (GCA_000956065.1), *Chlorocebus sabaeus* (GCA_000409795.2), *Macaca mulatta* (GCA_003339765.3), and *Pongo abelii* (GCA_002880775.3). The sequences were obtained from the Ensembl genome browser (46). The sequences from these species were aligned using Molecular Evolutionary Genetics Analysis software (MEGA, version 11, RRID:SCR_000667) [49]. The ClustalW algorithm was employed for multiple sequence alignment, and the conservation across the evaluated species and the human genome was visualized [50].

### 2.7 Gene ontology and pathway analyses

For each TF predicted to regulate *THY1*, we extracted the corresponding regulon (i.e., the set of genes inferred to be regulated by that TF) and subjected it to an overrepresentation analysis (ORA) using the clusterProfiler package (version 4.14.4; clusterProfiler, RRID:SCR_016884) [51]. Enrichment was assessed at a statistical threshold of p ≤ 0.05. Gene sets were obtained from the Molecular Signatures Database using the msigdbr package (version 7.5.1; msigdbr, RRID:SCR_022870) [52]. We focused on Gene Ontology Biological Processes (GOBPs), hallmark signatures of specific biological states, and curated collections from the Kyoto Encyclopedia of Genes and Genomes (KEGG), WikiPathways, and Reactome [[53], [54], [55], [56], [57]].

### 2.8 Acquisition of publicly available data for gastric cancer cell lines

We retrieved RNA-seq data from the Cancer Cell Line Encyclopedia (CCLE) to evaluate *THY1* expression levels in gastric cancer cell lines [58]. Cell lines lacking information on diffuse or intestinal subtypes were excluded. As with the patient samples, the data were normalized using the median-of-ratios (MRM) method and variance stabilizing transformation (VST) from the DESeq2 package (version 1.46.0; DESeq2, RRID:SCR_015687) [37].

### 2.9 Cell lines

The HGC-27 (diffuse type) and AGS (intestinal type) cell lines, authenticated by the Cell Bank of Rio de Janeiro (BCRJ), were cultured in DMEM (GIBCO Life Technologies, Carlsbad, CA, USA), whereas the KATO III (diffuse type) cell line, also authenticated by BCRJ, was cultured in IMDM (GIBCO Life Technologies, Carlsbad, CA, USA). The medium of HGC-27 and AGS cells was supplemented with 10 % fetal bovine serum (FBS; HyClone, USA), whereas the medium of KATO III cells were supplemented with 20 % FBS. All the cell lines were cultured in the presence of 100 IU/mL penicillin, 100 μg/mL streptomycin (Invitrogen, CA, USA), and 2 mM l-glutamine (Invitrogen, CA, USA).

### 2.10 Quantitative PCR (RT-qPCR)

For RT‒qPCR analyses, 2 μg of mRNA was treated with amplification-grade DNase I (Invitrogen, CA, USA) and reverse-transcribed using Superscript III Reverse Transcriptase® (Invitrogen, CA, USA). Each reaction contained 5 μL of SYBR Green PCR Master Mix® (Applied Biosystems, CA, USA), 2.5 μL of cDNA (corresponding to 10 ng of cDNA), and 2 μM of each primer. The reactions were performed in a Rotor-Gene 6000 thermocycler (Corbett, Australia). The cycling program consisted of a preincubation step at 95 °C for 5 min, followed by 45 cycles of 95 °C for 15 s and 62 °C for 40 s. A dissociation (melting) curve analysis confirmed that all the primers generated specific products with comparable amplification efficiencies. The relative fold change in expression was calculated using the 2^−ΔΔCt^ method, as described by Livak and Schmittgen [59]. Each sample was analyzed in triplicate, and B2M and GAPDH were used as internal reference genes. The primers used in this study are shown in Table 1.

**Table 1:** List of primers designed and used in this study.

Name	Sequence 5' — 3′	Strand	Size (bp)	Target
THY-1-F	ATCGCTCTCCTGCTAACAGTC	Foward	21	THY1
THY-1-R	CTCGTACTGGATGGGTGAACT	Reverse	21	THY1
THY1PX_I1_F	CTGCATAGCAACGTGAATGTATC	Foward	23	PRRX1 I1/I2 site
THY1PX_I1_R	GGGCTGAGGCTTCGTATTT	Reverse	19	PRRX1 I1/I2 site
THY1PX_P1_F	GCTTGGCAGACACAGAATTG	Foward	20	PRRX1 P1 site
THY1PX_P1_R	AGTGTTGCTGGGTGGAAG	Reverse	18	PRRX1 P1 site
THY1PX_P2_F	ACTCCGATCCTATCCACAGTAA	Foward	22	PRRX1 P2 site
THY1PX_P2_R	TGTGTCTGCCAAGCAAGAT	Reverse	19	PRRX1 P2 site
THY1TW_I1_F	GGATGGCGAGTGACTTAG	Foward	18	TWIST1 I1 site
THY1TW_I1_R	TCTCTCTGAATCTTACATACCC	Reverse	22	TWIST1 I1 site
THY1TW_I2_F	CCGCACAACATCTCAAACAAG	Foward	21	TWIST1 I2 site
THY1TW_I2_R	ACAATCAATGCTCTCTCTGTCC	Reverse	22	TWIST1 I2 site
THY1TW_P1_F	AAATGCCCTAGAAACCTCTG	Foward	20	TWIST1 P1 site
THY1TW_P1_R	AAGTGGGAGGAGATTCGCTTGG	Reverse	20	TWIST1 P1 site
THY1TW_P2_F	AGTGGGAAACGGAGCATC	Foward	18	TWIST1 P2 site
THY1TW_P2_R	GCTTCTGTCTGCCTCTTCAT	Reverse	20	TWIST1 P2 site
THY1TW_P3_F	ACTCCGATCCTATCCACAGTAA	Foward	22	TWIST1 P3 site
THY1TW_P3_R	TGTGTCTGCCAAGCAAGAT	Reverse	19	TWIST1 P3 site
THY1SN_P1_F	CTGGGTGCTCTGGAATAGATG	Foward	21	SNAI2 P1 site
THY1SN_P1_R	TCTGAAAGGAAGCTGGTTGG	Reverse	20	SNAI2 P1 site
THY1SN_P2_F	GAGTGAGAGAAAGTCATCAGCAC	Foward	23	SNAI2 P2 site
THY1SN_P2_R	ACTGAGTCGAGGCCAACT	Reverse	18	SNAI2 P2 site
THY1SN_P3_F	CATAAAGAGGCTGGCAGGA	Foward	19	SNAI2 P3 site
THY1SN_P3_R	TCCTGATAACCCACCGATTG	Reverse	20	SNAI2 P3 site
THY1SN_P4_F	GCCTCAAGTCCCAGCTATAAA	Foward	21	SNAI2 P4/P5 site
THY1SN_P4_R	AGGATTACTTGAGCCCAGGA	Reverse	20	SNAI2 P4/P5 site

### 2.11 Chromatin immunoprecipitation (ChIP) assays

We verified the potential binding of TWIST1, PRRX1 and SNAI2 to the *THY1* promoter region by using the SimpleChIP Enzymatic Chromatin IP Kit (Magnetic Beads) (Cell Signaling Technology, MA, USA) to perform ChIP assays according to the manufacturer's instructions. Briefly, chromatin from the HGC-27, AGS, and KATO III cell lines—which was previously prepared and digested with micrococcal nuclease—was incubated with 2 μg of either a TWIST1 antibody (sc-81417, Santa Cruz Biotechnology, TX, USA), a PRRX1 antibody (sc-293386, Santa Cruz Biotechnology, TX, USA), a SNAI2 antibody (#9585, Cell Signaling Technology, MA, USA) or a negative immunoprecipitation control consisting of a normal anti-IgG rabbit antibody (#2729, Cell Signaling Technology, MA, USA). In parallel, a 4 % fraction of each chromatin preparation was set aside as the input. The immunoprecipitated DNA was subsequently analyzed by qPCR for predicted binding sites using the primers described in the previous section. The percentage of input (% input) was calculated by normalizing the Ct values from the immunoprecipitated samples to the corresponding input sample. This normalization approach enabled the quantification of relative enrichment at each site across different conditions. For comparative analyses, we tested the significance of differences in % input values among the three cell lines using the Kruskal–Wallis test. When statistically significant differences were identified, we performed pairwise Mann–Whitney U tests, followed by Benjamini–Hochberg correction for multiple comparisons, to determine which specific pairs showed significant differences. These analyses were conducted independently for each predicted binding site.

### 2.12 Statistical analysis

All the statistical analyses were performed using R software (version 4.4.2, https://www.r-project.org/, accessed on January 10, 2025; R software, RRID:SCR_001905) [33]. For numeric variables, Student's *t*-test was applied when the data followed a normal distribution, whereas the Mann–Whitney *U* test was used for nonnormally distributed data. For comparisons involving categorical variables, Fisher's exact test was employed, as appropriate. p values ≤ 0.05 were considered statistically significant. When multiple comparisons were needed, the Benjamini–Hochberg method was used to adjust p values, and adjusted p values ≤ 0.05 were deemed statistically significant.

## 3 Results

### 3.1 Clinical and molecular characteristics of the cohorts

We collected gene expression data from 945 gastric cancer samples to delineate the regulatory landscape of gastric cancer and situate *THY1* within this context. We selected datasets spanning different gene expression platforms (RNA-seq and microarrays) and diverse clinicopathological characteristics to ensure robust and generalizable findings. After data acquisition, the samples were organized into three study cohorts based on the measurement methodology: TCGA cohort (n = 244, TCGA-STAD), the MA/Affymetrix cohort (n = 469, GSE15459, GSE38749, and GSE62254), and the MA/Illumina cohort (n = 232, GSE13861, GSE26899, and GSE26901).

This three-cohort design was chosen to minimize biases that could arise from relying on a single dataset or gene expression technology. By segregating the microarray data into Illumina and Affymetrix groups, we accounted for platform-specific differences in probe design and normalization pipelines. Moreover, incorporating multiple GSE studies within each microarray-based cohort reduced potential study-specific biases and strengthened the overall reliability of our analyses. All subsequent steps of the study were performed on these three cohorts. The main clinical and pathological features of the patients are described in detail in Table 2.

**Table 2:** Clinicopathological and demographic characteristics of the study cohorts.

Characteristics	TCGA cohort, N = 244^a^	MA/Affymetrix Cohort, N = 469^a^	MA/Illumina Cohort, N = 232^a^
**Sex**			
Female	88 (36 %)	165 (35 %)	68 (29 %)
Male	156 (64 %)	304 (65 %)	164 (71 %)

**Age**	67 (35–90)	64 (23–92)	59 (28–83)

**Tumor topology**			
Antrum	99 (42 %)	142 (52.4 %)	121 (52.1 %)
Body/Fundus	95 (40 %)	102 (37.6 %)	95 (41 %)
Cardia	44 (18 %)	27 (10 %)	11 (4.7 %)

**Lauren Class**			
Diffuse	64 (26 %)	220 (47 %)	72 (31 %)
Intestinal	180 (74 %)	249 (53 %)	160 (69 %)

**Pathological Stage**			
I	30 (13 %)	56 (11.9 %)	52 (22 %)
II	102 (44.1 %)	117 (25 %)	41 (18 %)
III	85 (36.8 %)	167 (35.6 %)	78 (34 %)
IV	14 (6.1 %)	129 (27.5 %)	61 (26 %)

a n (%); median (min —max). In this table, unavailable values have been omitted; therefore, percentage values have been calculated relative to the available data.

### 3.2 Comprehensive transcriptional network analysis

**Fig. 2:**
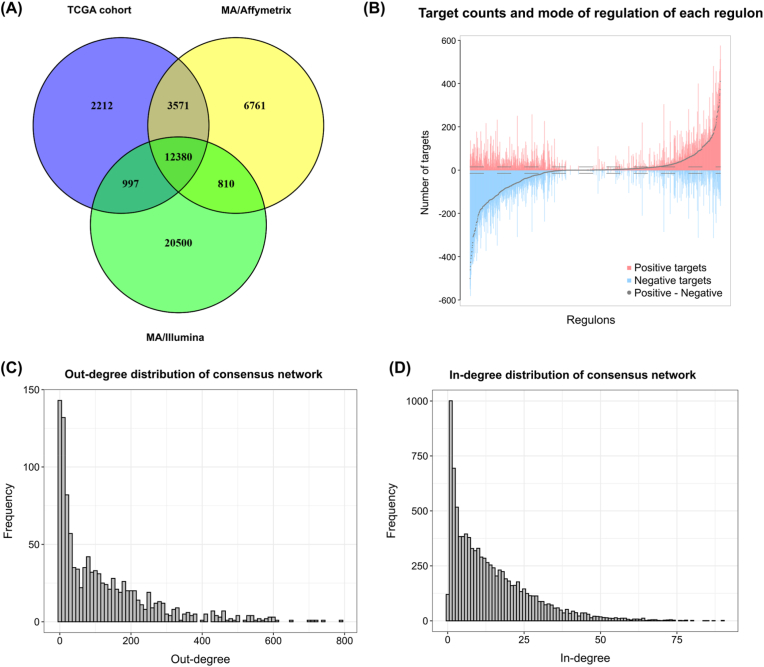
Transcriptional network construction and structural patterns. **(A)** Venn diagram showing the overlapping genes detected across the three cohorts' transcriptomic platforms. The central intersection of 12,380 genes represents those retained for network reconstruction. **(B)** For each regulon in the final consensus network, the numbers of positively regulated targets (red) and negatively regulated targets (blue) are plotted. The gray dots indicate the differences between the number of positive and negative targets (positive–negative). Each bar on the x-axis corresponds to a single regulon, whereas the y-axis shows the number of targets. The dashed horizontal lines mark the threshold of 15 targets. **(C)** Histogram depicting the out-degree distribution of transcription factors in the final consensus network. The x-axis shows the out-degree values (i.e., how many targets each TF regulates), and the y-axis indicates the frequency of each degree value. **(D)** Histogram illustrating the in-degree distribution of target genes in the final consensus network. The x-axis shows the in-degree values (i.e., how many TFs regulate each gene), and the y-axis indicates the frequency of each degree value.

We retained only those genes present across all the transcriptomic platforms in our three cohorts to ensure consistency and robustness in reconstructing the TRNs, yielding a final set of 12,380 genes (Fig. 2A). Among these genes, 1230 were annotated as transcription factors according to the AnimalTFDB and were thus designated potential regulators within the networks. Regulatory interactions were inferred via mutual information for each tumor subtype (diffuse and intestinal) in each cohort, resulting in six separate networks. After performing a permutation analysis with 1000 iterations in each network, we filtered out interactions with an adjusted p value below 3.28 × 10^−7^. We then conducted a bootstrap analysis (1000 iterations) and applied a data processing inequality (DPI) algorithm to remove indirect relationships. Next, we intersected the six resulting networks, retaining only those regulatory interactions that were consistent across all subtypes and cohorts. The final consensus network contained 9493 inferred targets, encompassing 134,997 regulatory connections and 1230 regulons (each consisting of a transcription factor and its predicted targets). For each regulatory connection, the mutual information value in the consensus network represents the average mutual information value across the individual networks. Specifically, 842 regulons included more than 15 inferred targets and were therefore considered more reliable. Of these, 797 presented more than 15 positively or negatively regulated targets, and 539 were classified as “balanced” regulons, each exhibiting at least 15 putative positive and 15 putative negative targets (Fig. 2B).

We observed that the out-degree distribution followed a skewed pattern typical of biological networks, with a median of 87.5 and a maximum of 793 predicted targets per TF (Fig. 2C). Similarly, the in-degree distribution also showed a skewed profile, with a median of 10 and a maximum of 90 predicted regulators per target (Fig. 2D). In addition, the network exhibited a scale-free-like topology, as indicated by the Kolmogorov–Smirnov test for adequacy to a power-law distribution for both the out-degree (p = 0.07) and in-degree (p = 0.06) profiles. This pattern underscores a hierarchical regulatory structure characteristic of biological networks, with a few key transcription factors acting as major hubs. These hubs are putative master regulators based on their high connectivity and conserved presence across datasets, a hallmark of key regulatory nodes in biological networks. Moreover, the comprehensive construction of the transcriptional network provided a global framework to identify potential master regulators, setting the stage for targeted analyses of THY1 and its regulatory landscape.

### 3.3 Comprehensive refinement identifies six transcription factors as robust regulators of THY1 expression through network inference and binding site prediction

**Fig. 3:**
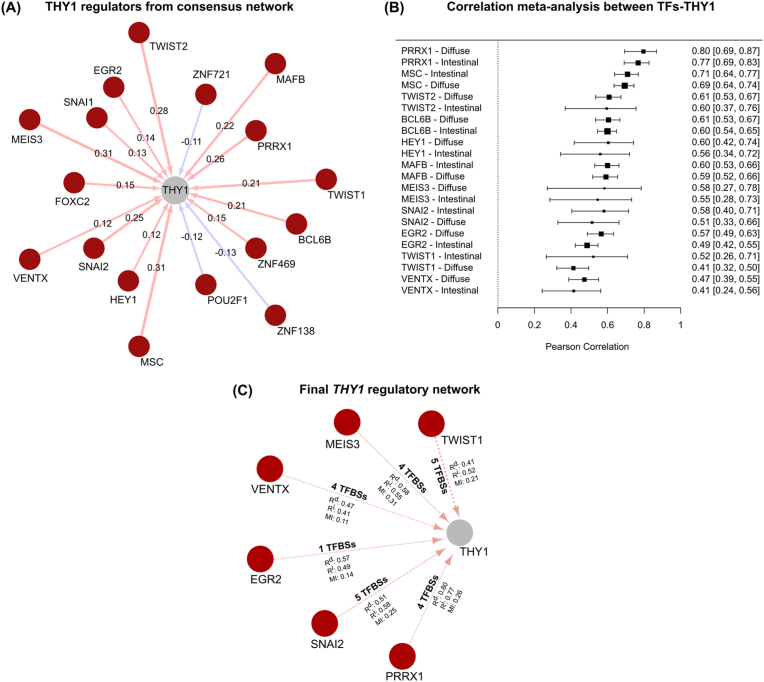
Identification and refinement of putative *THY1* regulators. **(A)** Network representation of putative *THY1* regulators derived from the final consensus network. Transcription factors (TFs) are shown in dark red, and *THY1* is shown in gray. Light red edges indicate predicted positive regulation, whereas blue edges indicate predicted negative regulation. The numerical values on the edges represent the mutual information (MI), and the width of each edge is scaled according to the absolute MI value. **(B)** Forest plot illustrating the Pearson correlation meta-analysis between each TF and *THY1* in the intestinal and diffuse subtypes. TFs are listed in descending order of aggregated correlation magnitude. Only TF–THY1 correlations meeting the significance threshold (p < 0.05) and |R| > 0.4 are displayed. The x-axis shows the combined correlation coefficient obtained using a random-effects model, and the dotted vertical line indicates R = 0. Each TF is listed for both subtypes, with black squares representing the combined correlation estimate and horizontal lines denoting the 95 % confidence interval. The corresponding numeric values (aggregate correlation and 95 % confidence interval) are shown on the right. **(C)** Network representation of the final regulatory network of *THY1* after all refinement steps. TFs are shown in dark red, and *THY1* is shown in gray. The number of transcription factor binding sites (TFBSs) for each regulator is indicated on the edge connecting the TF to *THY1*. Additionally, the numeric values on each edge represent the combined correlation estimates for the diffuse subtype (R^d^) and the intestinal subtype (R^i^), as well as the mutual information (MI) between the TF and *THY1*.

Building on the comprehensive transcriptional network described above, we next focused on identifying and refining the subset of putative regulators targeting *THY1*. A total of 17 regulators were identified in the consensus network, including 14 predicted positive regulators and 3 negative regulators. According to the absolute values of mutual information, the top 10 regulators were MEIS3 (0.313), MSC (0.306), TWIST2 (0.281), PRRX1 (0.262), SNAI2 (0.252), MAFB (0.220), BCL6B (0.212), TWIST1 (0.212), ZNF469 (0.153), and FOXC2 (0.151), as summarized in Table 3. A network representation of these regulators is shown in Fig. 3A.

**Table 3:** Putative regulators of the *THY1* gene in the consensus transcriptional network.

Transcription Factor	Description	Target	Weight^a^
MEIS3	Meis homeobox 3	THY1	0.313
MSC	musculin	THY1	0.306
TWIST2	twist family bHLH transcription factor 2	THY1	0.281
PRRX1	paired related homeobox 1	THY1	0.262
SNAI2	snail family transcriptional repressor 2	THY1	0.252
MAFB	MAF bZIP transcription factor B	THY1	0.220
BCL6B	BCL6B transcription repressor	THY1	0.212
TWIST1	twist family bHLH transcription factor 1	THY1	0.212
ZNF469	zinc finger protein 469	THY1	0.153
FOXC2	forkhead box C2	THY1	0.151
EGR2	early growth response 2	THY1	0.137
SNAI1	snail family transcriptional repressor 1	THY1	0.133
HEY1	hes related family bHLH transcription factor with YRPW motif 1	THY1	0.123
VENTX	VENT homeobox	THY1	0.115
ZNF721	zinc finger protein 721	THY1	−0.112
POU2F1	POU class 2 homeobox 1	THY1	−0.123
ZNF138	zinc finger protein 138	THY1	−0.132

a The weight represents the mutual information value, while the sign indicates the predicted mode of action based on Pearson's correlation coefficient between the regulator and its targets.

We performed a Pearson correlation meta-analysis between *THY1* and each of the 17 putative regulators, considering both tumor subtypes (diffuse and intestinal) across the three cohorts, to obtain deeper insights into these candidate regulators. For each transcription factor (TF)–THY1–subtype combination, correlation coefficients were calculated for the three cohorts and then aggregated using a random-effects model, resulting in a total of 34 meta-analyses (17 TFs × 2 subtypes). We applied a significance threshold (p < 0.05) and a minimum absolute correlation (|R| > 0.4) for both subtypes, retaining only those TFs that met these criteria in both subtypes. This filtering step yielded 11 TFs, corresponding to 22 relevant meta-analyses (11 TFs × 2 subtypes). Overall, the 11 TFs that satisfied these criteria, listed in descending order of correlation magnitude, were PRRX1, MSC, TWIST2, BCL6B, HEY1, MAFB, MEIS3, SNAI2, EGR2, TWIST1, and VENTX (Fig. 3B).

We searched for consensus binding sites for these transcription factors within the *THY1* promoter region (3 kb upstream of the transcription start site) and the first intron (2186 bp) to refine this list further and strengthen the evidence for their regulatory roles. Consensus binding site motifs were obtained from the JASPAR database. Regulators for which no binding site data were available, such as MAFB and TWIST2, were excluded from this analysis. For the remaining nine regulators, we scanned the designated regulatory regions for binding sites using a minimum score threshold of 85 % and retained only canonical binding sites when this information was available.

As a result, six of the nine evaluated regulators had binding sites within the *THY1* regulatory regions that met our criteria. SNAI2 had five sites (all in the promoter), TWIST1 had five sites (three in the promoter and two in the intron), PRRX1 had four binding sites (two in the promoter and two in the intron), MEIS3 had four sites (all in the promoter), VENTX had four sites (two in the promoter and two in the intron), and EGR2 had one site (in the promoter). These findings provide robust evidence for the potential direct regulatory effects of these transcription factors on *THY1*, forming the final regulatory network of *THY1* (Fig. 3C).

This integrative approach underscores the robustness of our methodology, which combines statistical correlations with mechanistic predictions to pinpoint transcription factors likely to directly regulate *THY1* expression. By incorporating both network-level insights and the regulatory sequence analysis, we provide a multifaceted view of the regulatory framework surrounding *THY1.* The refined list of six transcription factors provides a robust foundation for exploring the biological roles of these regulators in gastric cancer. We next examined the functional relevance of these factors through an enrichment analysis of their regulons to gain additional insights.

### 3.4 The functional enrichment analysis highlights THY1 as part of a transcriptional program driving the EMT and ECM remodeling in gastric cancer

Building on the identification of six transcription factors (TFs) as robust putative regulators of *THY1*, we sought to explore the biological pathways and processes associated with their regulons. These regulons, defined as the sets of genes inferred to be regulated by each TF in the global consensus network, varied in size. Specifically, the regulon sizes for the six putative regulators were as follows: EGR2 (374 targets), VENTX (358 targets), MEIS3 (279 targets), PRRX1 (178 targets), SNAI2 (120 targets), and TWIST1 (92 targets).

**Fig. 4:**
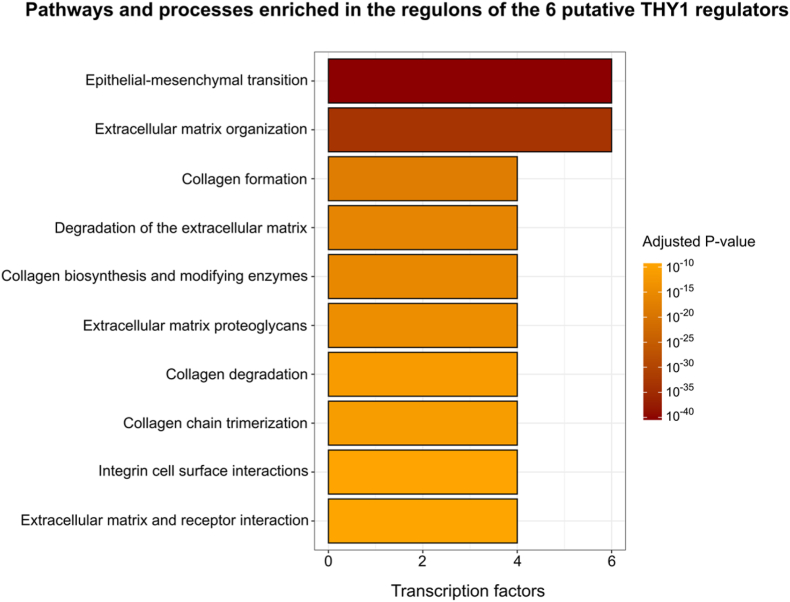
Pathways and biological processes enriched in the regulons of the six putative *THY1* regulators. The x-axis represents the number of transcription factors whose regulons were significantly enriched for the evaluated signaling pathways and biological processes. The y-axis lists the names of the enriched processes, which are organized in descending order of significance, with the most significant process appearing at the top. The color gradient corresponds to the adjusted *p* value (Benjamini–Hochberg correction), with orange indicating higher values and dark red representing lower and more significant values.

For the functional enrichment analysis of these regulons, we used a reference collection of gene sets from the GOBP, Hallmarks, KEGG, WikiPathways, and Reactome databases. Pathways and processes with a corrected p value ≤ 0.05 were considered significantly enriched. The results revealed that the epithelial–mesenchymal transition (EMT) was the most significantly enriched process (p = 3.3 × 10^−41^) and was present in the regulons of all six TFs (Fig. 4). Similarly, extracellular matrix organization was another highly enriched process shared across all six regulons. Additionally, several other processes related to extracellular matrix (ECM) remodeling or cell–matrix interactions were among the top 10 enriched pathways. These processes included collagen formation, degradation of the extracellular matrix, collagen biosynthesis and modifying enzymes, extracellular matrix proteoglycans, collagen degradation, collagen chain trimerization, integrin cell surface interactions, and extracellular matrix–receptor interactions.

These findings support the hypothesis that *THY1* may be an integral component of the epithelial-mesenchymal transition transcriptional program in the context of gastric cancer. The six inferred regulators orchestrate key pathways involved in ECM remodeling and cell–matrix interactions, processes fundamental for tumor progression and metastasis. These insights guided the selection of experimental models to further validate the regulatory interactions predicted *in silico*.

### 3.5 Converging evidence from public data and experimental studies highlights the heterogeneity of THY1 expression in gastric cancer cell lines

Given the robust bioinformatic evidence supporting the regulation of *THY1* as a component of an EMT transcriptional program in gastric cancer, we next sought to establish an experimental model to validate the regulation of *THY1* by its putative transcriptional regulators. We first examined publicly available gene expression data from gastric cancer cell lines to determine whether the heterogeneity of *THY1* expression observed in patients’ tumors is also reflected in these *in vitro* models and to translate the bioinformatic findings into experimental evidence.

**Fig. 5:**
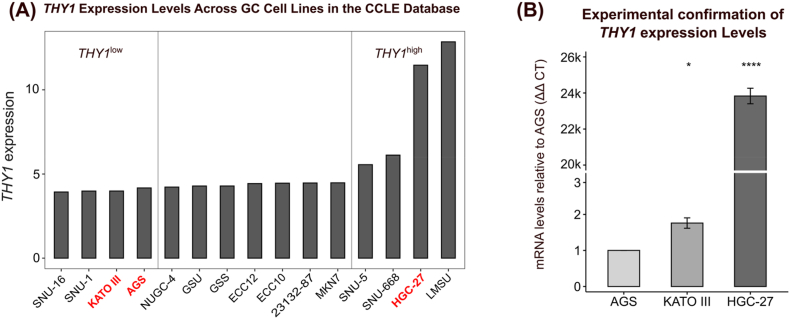
Heterogeneity in *THY1* expression in gastric cancer cell lines. **(A)** Bar graph showing the expression levels of *THY1* across gastric cancer cell lines based on CCLE data. The cell lines are arranged from the lowest to the highest *THY1* expression levels. The vertical gray lines indicate the lower and upper quartiles of expression, defining the *THY1*^low^ and *THY1*^high^ groups, respectively. The cell lines highlighted in red were selected for experimental validation because of their availability in our laboratory. The expression levels are reported as variance-stabilized transformation (VST) values. **(B)** Bar graph comparing the relative *THY1* mRNA expression levels among the HGC-27, KATO III, and AGS cell lines, as determined by RT‒qPCR. A break in the y-axis was introduced to visualize differences across cell lines with distinct expression levels better. All the experiments were conducted in triplicate, and the AGS cell line was used as a reference for comparison. The data are shown as the means ± standard deviations. ns: not significant, ∗p < 0.05, ∗∗p < 0.01, ∗∗∗p < 0.001, and ∗∗∗∗p < 0.0001.

After obtaining RNA-seq data for gastric cancer cell lines from the CCLE database, we filtered the dataset to include only cell lines with available histological subtype information (diffuse or intestinal), resulting in 15 cell lines being evaluated. The data were preprocessed and normalized with the same procedure used for patients’ RNA-seq samples in previous analyses. The cell lines were subsequently ranked by *THY1* expression levels and categorized into quartiles, with the lower quartile designated *THY1*^low^ and the upper quartile designated *THY1*^high^. This analysis revealed substantial heterogeneity in *THY1* expression across the evaluated cell lines (Fig. 5A).

Given the lack of replicates for individual cell lines in the CCLE dataset, which limits the statistical power, and to confirm the trends observed in the public data, we selected representative cell lines from the *THY1*^high^ and *THY1*^low^ groups that were available in our laboratory: KATO III and AGS cells for the *THY1*^low^ group and HGC-27 cells for the *THY1*^high^ group. Each cell line was cultured in triplicate to ensure robust experimental validation, and RT‒qPCR was performed to measure *THY1* expression, with AGS (low expression in the public dataset) used as the reference cell line. Consistent with the RNA-seq results, the HGC-27 cell line exhibited significantly higher expression of *THY1* than AGS cells (p < 0.001, Fig. 5B).

These findings confirm the heterogeneity of *THY1* expression in gastric cancer cell lines, mirroring the patterns observed in patients’ tumors. By integrating public data with internally performed experiments, we established a robust model for investigating the transcriptional regulation of *THY1*, ensuring the translational relevance of our bioinformatic predictions.

### 3.6 Experimental evidence supporting TWIST1 and SNAI2 as central regulators of THY1 expression in gastric cancer

Having established an experimental model of gastric cancer cell lines with high and low *THY1* expression, we next aimed to validate the direct regulation of *THY1* by its putative transcriptional regulators. Among the six robustly identified regulators, PRRX1, TWIST1, and SNAI2 were selected for experimental validation based on their strong bioinformatic evidence, including the number of predicted binding sites within the *THY1* regulatory regions. We assessed the conservation of their binding sites across closely related species to further prioritize these regulators, as conservation in noncoding regions often reflects functional importance.

**Fig. 6:**
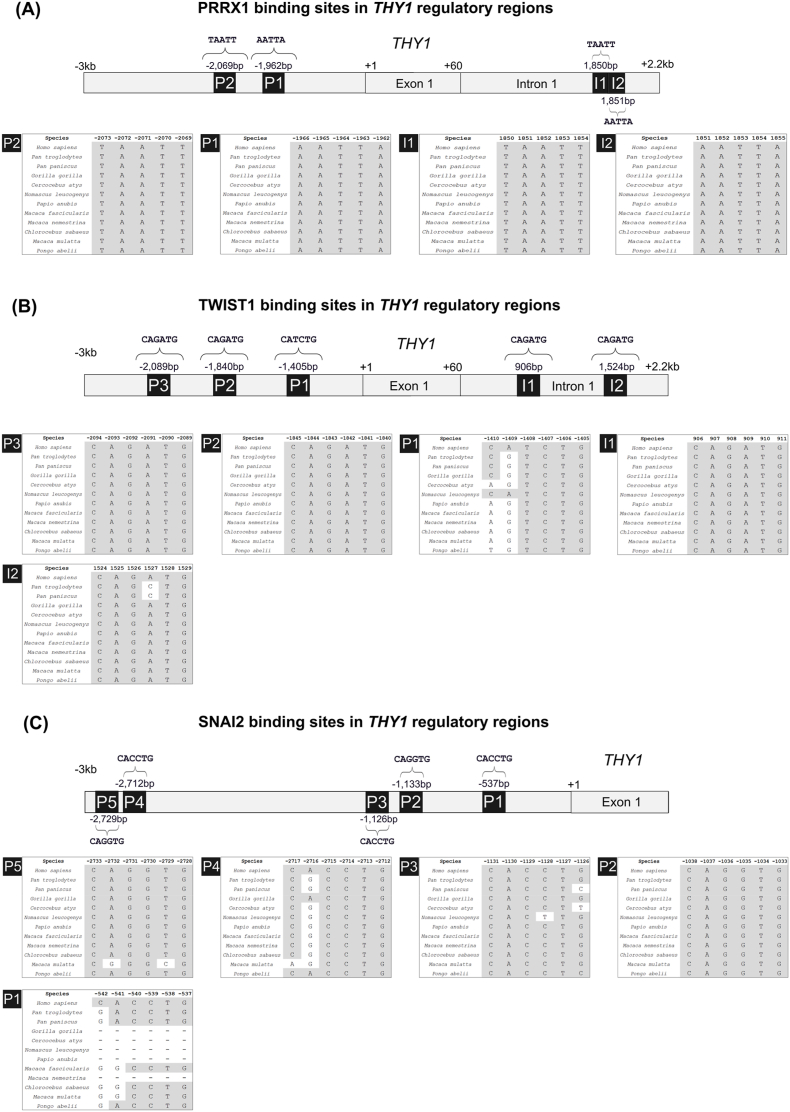
Conservation of PRRX1, TWIST1, and SNAI2 binding sites in *THY1* regulatory regions. The figure shows the location and conservation of transcription factor binding sites within the *THY1* regulatory regions for PRRX1 **(A)**, TWIST1 **(B)**, and SNAI2 **(C)**. At the top of each panel, a schematic representation of the *THY1* gene is shown, where +1 marks the transcription start site, and the 3-kb upstream region is defined as the promoter. The first intron begins after the end of exon 1 (+60). Binding sites are labeled with their respective names, and their positions relative to the transcription start site are indicated. Below each schematic, alignment tables show the conservation of binding sites across the evaluated species. Conserved nucleotides are highlighted in gray, whereas nonconserved nucleotides are highlighted in white. The species names are listed on the left, and the nucleotide positions relative to the human sequence are shown above each alignment. The schematics of the gene structure, regulatory regions, and binding site positions are illustrative and not drawn to scale.

The conservation analysis revealed varying levels of conservation for the predicted binding sites of PRRX1, TWIST1, and SNAI2. For PRRX1, four binding sites were identified (two in the promoter and two in the intron), all of which were 100 % conserved across the evaluated species (Fig. 6A). TWIST1 exhibited five predicted binding sites (three in the promoter and two in the intron), three of which (I1, P2, and P3) were fully conserved across all the species (Fig. 6B). However, I2 displayed divergence in two species, and P1 showed variability at one position in seven species and at another position in 10 species. For SNAI2, all five predicted binding sites were located in the promoter region, but only P2 demonstrated complete conservation across species (Fig. 6C). P1, located in a region with alignment gaps, exhibited divergence in five species at certain positions and in all 11 species at another position. P3, P4, and P5 showed varying levels of divergence, with P5 displaying the highest conservation among these sites.

Following the conservation analysis, we performed ChIP assays for PRRX1, TWIST1, and SNAI2 in the HGC-27 (*THY1*^high^), AGS, and KATO III (*THY1*^low^) cell lines. Primers were designed for the binding site. ChIP‒qPCR was then conducted to assess binding activity. The percent input values were calculated, and the values of the IgG control were subtracted to normalize the results.

**Fig. 7:**
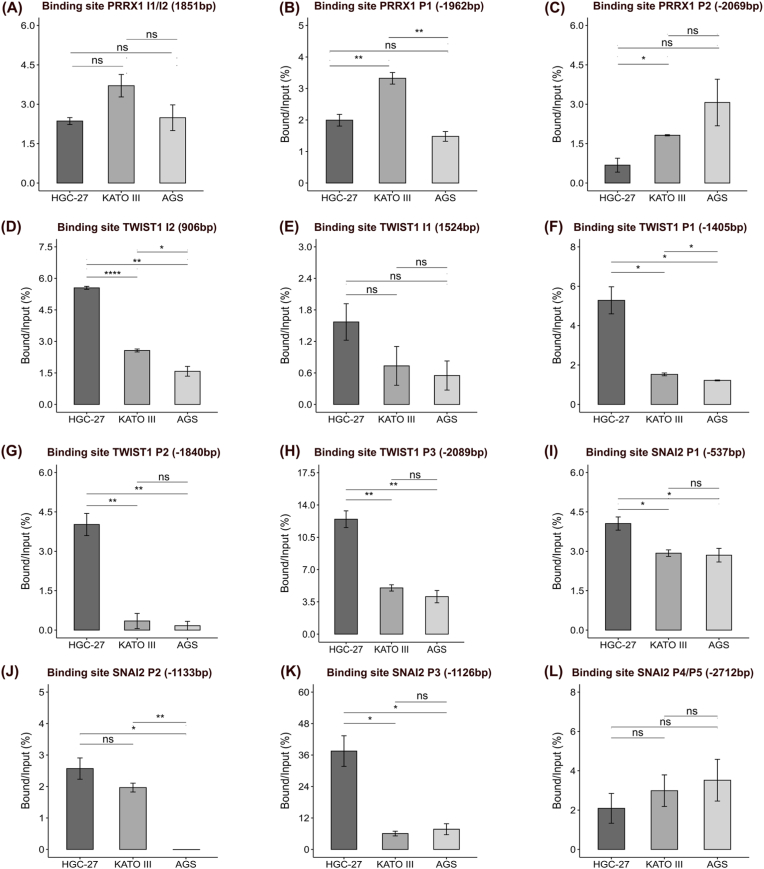
Chromatin immunoprecipitation analysis of PRRX1, TWIST1 and SNAI2 binding sites in *THY1* regulatory regions across gastric cancer cell lines. The bar graphs represent the bound/input (%) values for each binding site evaluated. The y-axis indicates the percentage of DNA recovered (bound) relative to the input DNA, and the x-axis shows the cell lines analyzed: HGC-27 (*THY1*^high^), Kato III, and AGS (both *THY1*^low^). The title of each panel specifies the transcription factor, the binding site name, and its position within the *THY1* regulatory regions. **(A**–**C)** correspond to PRRX1 binding sites, **(D**–**H)** correspond to TWIST1 binding sites, and **(I**–**L)** correspond to SNAI2 binding sites. The error bars represent the standard deviations of three replicates. Statistical significance was determined using pairwise Mann‒Whitney U tests with p value correction. For each bound/input (%) analysis, the bound/input (%) value of the control IgG was subtracted from the value of each cell line. The data are shown as the means ± standard deviations. ns: not significant, ∗p < 0.05, ∗∗p < 0.01, ∗∗∗p < 0.001, and ∗∗∗∗p < 0.0001.

The analysis of PRRX1 binding revealed significant but minor differences at specific sites. While the I1/I2 site showed no significant variation across cell lines (Fig. 7A), the P1 site was significantly enriched in KATO III cells relative to the other cell lines, and the P2 site was significantly enriched in KATO III cells compared with HGC-27 cells (Fig. 7B). However, these differences were considerably small (a maximum difference of 1.84 percentage points for P1 and 1.14 for P2), suggesting that while PRRX1 may bind these regions, the magnitude of these differences implies a limited impact on differential *THY1* expression in these cell lines. TWIST1 showed greater variability in binding activity across sites and cell lines. Compared with the other cell lines, HGC-27 cells exhibited significantly greater binding at the TWIST1 I2 site (Fig. 7D). However, at the TWIST1 I1 site (Fig. 7E), no significant differences in binding were observed. Furthermore, at the TWIST1 P1 and P2 sites (Fig. 7F and G), significantly higher binding activity was observed in HGC-27 cells than in both AGS and KATO III cells (p < 0.05). Finally, the TWIST1 P3 site (Fig. 7H) showed the greatest difference in binding, with significantly higher activity observed in HGC-27 cells (12.5 % input) than in KATO III (5.01 %) and AGS (4.06 %), representing an approximately 2.5-fold increase over KATO III and a 3.1-fold increase over AGS (150–210 % relative increase; p < 0.05). Interestingly, the TWIST1 P2 and P3 binding sites, which were 100 % conserved across all the analyzed species (Fig. 6B), also demonstrated significant binding activity in HGC-27 cells, suggesting their functional importance in regulating *THY1* expression. Conversely, the P1 site, which diverged across multiple species, still exhibited differential binding, highlighting the complexity of *THY1* regulatory interactions. SNAI2, like TWIST1, showed significant variability in binding activity across different sites and cell lines. At the SNAI2 P1 site (Fig. 7I), significantly higher binding activity was observed in HGC-27 cells than in both AGS and KATO III cells (p < 0.05), whereas at the SNAI2 P2 site (Fig. 7J), higher binding activity was observed in HGC-27 and KATO III cells than in AGS cells. Additionally, the SNAI2 P3 site (Fig. 7K) presented the greatest difference in binding among all the sites evaluated, with significantly higher activity observed in HGC-27 cells (37.5 % input) than in both KATO III (6.05 %) and AGS (7.71 %) corresponding to a 6.2-fold increase over KATO III and a 4.9-fold increase over AGS (>500 % relative increase; p < 0.05). Finally, at the SNAI2 P4/P5 site (Fig. 7L), no significant difference was observed. This co-occurrence of high TWIST1 and SNAI2 binding in HGC-27 cells raises the possibility of coordinated regulation by these two transcription factors. Moreover, additional functional assays are needed to establish this relationship.

Overall, these results suggest that TWIST1 and SNAI2 likely play prominent roles in the regulation of *THY1* expression in the studied gastric cancer cell lines, with specific binding sites (I2, P1, P2, and P3 for TWIST1 and P1, P2, and P3 for SNAI2) in the *THY1* regulatory regions contributing to its differential expression. In contrast, PRRX1 binding, while present, showed less pronounced differences between the cell lines, suggesting a potentially less direct or less impactful role. Together, these findings provide experimental evidence supporting TWIST1 and SNAI2 as direct regulators of *THY1*, integrating bioinformatic predictions and experimental insights into a cohesive model of *THY1* transcriptional regulation in the context of gastric cancer.

## 4 Discussion

This study comprehensively integrated diverse transcriptomic datasets spanning multiple gene expression platforms and clinical characteristics to construct a robust transcriptional regulatory network in gastric cancer. Focusing on the *THY1* gene, a known marker of a poor prognosis for patients with gastric cancer, we employed a rigorous multistep refinement process [[8], [9], [10], [11], [12]]. This approach narrowed the 1230 TFs in the network to six putative regulators of *THY1*: PRRX1, TWIST1, SNAI2, MEIS3, VENTX, and EGR2. By combining consensus network construction, robust correlation meta-analyses, and *in silico* TFBS predictions, we ensured consistency across platforms, histological subtypes, and cohorts. These findings underscore the robustness of the inferred regulatory relationships and provide a strong foundation for understanding the complex regulation of *THY1*. TRNs play a critical role in orchestrating gene expression programs, particularly in cancer, where they govern processes linked to tumor progression and the prognosis [60,61]. While previous studies have explored TRNs in gastric cancer, they have largely focused on broad regulatory changes rather than dissecting the regulation of specific genes. In this context, Xu et al. (2016) identified 70 TFs with altered activity that regulate approximately 566 target genes, elucidating pathways involved in gastric tumorigenesis [62]. Similarly, Yu et al. (2022) described the use of transcriptional networks to identify experimentally testable regulatory mechanisms [63]. However, studies that focus specifically on elucidating the regulatory landscape of key prognostic genes such as *THY1* remain scarce. Moreover, many network inference studies rely on single datasets or transcriptomic platforms, limiting their generalizability across tumor contexts. By integrating multiple datasets and platforms, our study not only addresses these limitations but also highlights the potential of such approaches to identify robust and biologically meaningful regulatory mechanisms. This methodological rigor is particularly critical in understanding the transcriptional complexity of genes such as *THY1*, whose expression is highly context dependent and associated with aggressive tumor phenotypes.

Building on the identification of six robust regulators of *THY1*, our functional enrichment analysis of their regulons revealed that all six transcription factors orchestrate gene expression patterns associated with the epithelial–mesenchymal transition (EMT) and extracellular matrix (ECM) organization. The EMT is a fundamental biological process in which epithelial cells acquire mesenchymal characteristics, lose their cell–cell adhesion ability and gain migratory and invasive capabilities [64]. In cancer, this process is widely recognized as a key driver of tumor progression and metastasis [65]. Similarly, ECM organization plays a critical role in establishing a tumor-permissive microenvironment, facilitating not only tumorigenesis but also invasion and metastatic spread [66]. These processes are intricately linked, as ECM-derived signals are crucial for initiating and sustaining the EMT during tumor progression, whereas the EMT process itself drives ECM remodeling to support tumor progression [67,68]. Consistent with these findings, several of the putative *THY1* regulators identified in this study, including PRRX1, SNAI2, TWIST1, and MEIS3, have previously been implicated in regulating the EMT and ECM remodeling in cancer [[69], [70], [71], [72], [73]]. Notably, our group has also shown in prior work that the EMT and matrix remodeling are hallmark processes characterizing gastric tumors with high *THY1* expression [12]. Similarly, Xue et al. (2011) showed that gastric cancer stem cells expressing *THY1* exhibit mesenchymal features and high expression of EMT markers such as TWIST1 [74]. Additionally, *THY1* itself has been described as a regulator of cell–matrix interactions, reinforcing its role in modulating the tumor microenvironment [75]. Taken together, these findings suggest that *THY1* is not merely a biomarker of the EMT process but may also be an integral component of the EMT transcriptional program in gastric cancer. This dual role as both a marker of and a potential participant in the EMT may offer a biological explanation for the association between high *THY1* expression and a poor prognosis for patients with gastric tumors.

We investigated the regulation of *THY1* expression by its putative transcriptional regulators in gastric cancer cell lines to translate our bioinformatic predictions into experimental evidence. Among the six identified regulators, PRRX1, TWIST1, and SNAI2 were prioritized for our experiments based on their robust bioinformatic data and strong associations with EMT pathways. After confirming the *THY1* expression pattern in cell lines—HGC-27 (*THY1*^high^) and KATO III and AGS (*THY1*^low^)—we integrated conservation analyses of binding sites with chromatin immunoprecipitation assays to assess direct regulatory interactions. Our findings revealed that TWIST1 and SNAI2 play prominent roles as direct regulators of *THY1*, with significant binding activity observed in the HGC-27 cell line, whereas *PRRX1* exhibited less pronounced differences across the evaluated cell lines, suggesting a potential role in the basal regulation of *THY1*. Prior studies have highlighted the evolutionary divergence of *THY1* regulatory elements between humans and model organisms, complicating the development of *in vivo* models that accurately reflect human *THY1* regulation [26]. Moreover, *THY1* expression is governed by distinct regulatory regions that vary across tissues, with the deletions of these regions selectively impacting expression in some tissues while sparing others [[27], [28], [29]]. These findings underscore the importance of identifying *THY1* regulators in the specific context of gastric cancer. Although the transcriptional regulation of *THY1* in cancer remains largely unexplored, Lu et al. (2014) identified a population of breast cancer stem cells characterized by high *THY1* expression and EMT properties, indicating that the induction of both TWIST1 and SNAI2 in breast cell lines upregulated *THY1* expression [76]. This result aligns with our findings, as the HGC-27 cell line, which exhibits high *THY1* expression and significant TWIST1 and SNAI2 binding at its promoter, has also been reported to display cancer stem cell characteristics [22,77]. Similarly, Jiang et al. (2011) reported that cancer stem cells expressing *THY1* can resist conventional treatments and reestablish the tumor hierarchy [25]. These findings address a key question in the context of gastric cancer: what activates *THY1* expression in aggressive tumors, and what does this activation signify biologically? By identifying TWIST1 and SNAI2 as potential direct regulators of *THY1* and linking them to the EMT transcriptional program, we provide a foundational step toward answering this question. These findings not only provide insights into the mechanisms that may govern *THY1* expression but also support a potential connection between its role as a prognostic marker and its involvement in tumor progression and the EMT, paving the way for further research into its biological and clinical significance.

In another aspect of *THY1* regulation in the context of gastric cancer, recent studies have also proposed complementary hypotheses involving distal regulatory elements. Notably, data from Razavi-Mohseni et al. (2024) showed a Mes-like enhancer located approximately 10 kb upstream of the *THY1* promoter, marked by strong ATAC-seq signal in LMSU cells, one of the gastric cancer lines with high *THY1* expression and mesenchymal characteristics [78]. This enhancer is part of a CTCF-defined chromatin loop that includes the *THY1* promoter and has been shown to be bound by transcription factors such as JUN and RUNX2 in other cell types, suggesting a potential role for enhancer–promoter interactions in *THY1* regulation [79]. While our study focused on proximal promoter and intronic regions to identify transcriptional regulators, these findings underscore the regulatory complexity of *THY1* and the possibility that distal enhancers cooperate with promoter-bound TFs to drive its expression. Importantly, although our regulatory network was inferred from transcriptomic data in patient cohorts, not chromatin accessibility profile, some of the regulators highlighted in the enhancer study, such as TWIST1 and SNAI2, also emerged as key players in our analysis, suggesting convergence across regulatory layers. Furthermore, multiple regulatory mechanisms, potentially involving various transcription factors such as JUN and RUNX2, may act in combination to control *THY1* expression, which aligns with its well-recognized context-dependent behavior. Our findings provide a transcriptional framework that can be further explored in the context of enhancer activity. This enhancer-based regulatory model presents a relevant complementary mechanism for the integration of transcriptomic and epigenomic data to fully elucidate the multilayered regulation of *THY1* in gastric cancer.

While our study provides novel insights into the transcriptional regulation of *THY1* in gastric cancer, several limitations should be acknowledged. Although the integration of bioinformatic predictions with experimental evidence strengthens the validity of our findings, the study is inherently limited by the *in vitro* nature of the experimental models. Gastric cancer is a highly heterogeneous disease, and the regulation of *THY1* expression may vary depending on the tumor microenvironment or additional epigenetic factors not addressed in this study. Furthermore, while we showed that TWIST1 and SNAI2 are potential direct regulators of *THY1*, the precise dynamics of this interaction under different conditions, as well as potential coregulatory mechanisms, merit further exploration. Despite these limitations, our work represents a critical step toward deciphering the transcriptional complexity of *THY1*, particularly in the context of gastric cancer. This study is among the first to systematically delineate the regulatory network governing *THY1* expression, identifying robust candidate regulators through rigorous bioinformatic and experimental analyses. By integrating a high-dimensional data analysis with experimental validation, we highlight the synergy between bioinformatics and benchwork in uncovering testable and biologically relevant mechanisms. Ultimately, our findings enhance the understanding of *THY1* as a key player in the EMT and gastric cancer progression, providing a potential biological explanation for its association with a poor prognosis. Furthermore, this approach provides a framework for investigating the regulatory landscapes of other prognostic markers in cancer, paving the way for the development of targeted therapeutic strategies.

## 5 Conclusions

In this study, we systematically delineated the transcriptional regulatory landscape of *THY1* in gastric cancer by identifying six robust putative regulators—PRRX1, TWIST1, SNAI2, MEIS3, VENTX, and EGR2—through an integrative multicohort approach. The functional enrichment analysis revealed that these regulators could orchestrate transcriptional programs associated with the epithelial–mesenchymal transition (EMT) and extracellular matrix (ECM) remodeling, supporting the hypothesis that *THY1* is not only a marker but also potentially an integral component of the EMT transcriptional program. This study provides experimental evidence of TWIST1 and SNAI2 binding to the *THY1* regulatory regions in a gastric cancer cell line with high *THY1* expression, supporting the hypothesis that these transcription factors may act as key regulators of THY1 expression in this context. These findings suggest a mechanistic link between *THY1* expression and EMT-driven transcriptional regulation, offering a biological explanation for the association of high *THY1* expression with a poor prognosis for gastric cancer patients. Our work represents a crucial step in elucidating the transcriptional complexity of *THY1* and underscores the power of integrating bioinformatic predictions with experimental validation to elucidate fundamental regulatory mechanisms in tumor biology.

## CRediT authorship contribution statement

**Paulo Rohan:** Writing – review & editing, Writing – original draft, Visualization, Validation, Software, Methodology, Investigation, Formal analysis, Data curation, Conceptualization. **Everton Cruz dos Santos:** Writing – review & editing, Writing – original draft, Visualization, Validation, Methodology, Investigation, Formal analysis. **Pedro Leite Azevedo:** Writing – review & editing, Writing – original draft, Visualization, Validation, Methodology, Investigation, Formal analysis. **Jessica Oliveira da Conceição:** Writing – review & editing, Writing – original draft, Visualization, Validation, Methodology, Investigation, Formal analysis. **Eliana Abdelhay:** Writing – review & editing, Writing – original draft, Supervision, Resources, Project administration, Funding acquisition, Conceptualization. **Renata Binato:** Writing – review & editing, Writing – original draft, Supervision, Resources, Project administration, Funding acquisition, Conceptualization.

## Ethical approval and consent to participate

Not applicable.

## Consent for publication

Not applicable.

## Data availability statement

The raw data can be obtained from online databases, including TCGA database (https://portal.gdc.cancer.gov/) under study abbreviation TCGA-STAD and the GEO database (http://www.ncbi.nlm.nih.gov/geo) using the following accession numbers: GSE13861, GSE15459, GSE26899, GSE26901, GSE38749 and GSE62254.

## Funding

This work was supported by a grant from the 10.13039/501100004586Fundação de Amparo à Pesquisa do Estado do Rio de Janeiro (FAPERJ) [grant number SEI-260003/001147/2020].

## Declaration of competing interest

The authors declare that they have no known competing financial interests or personal relationships that could have appeared to influence the work reported in this paper.
